# Integrin α_D_β_2_ (CD11d/CD18) Modulates Leukocyte Accumulation, Pathogen Clearance, and Pyroptosis in Experimental *Salmonella* Typhimurium Infection

**DOI:** 10.3389/fimmu.2018.01128

**Published:** 2018-05-24

**Authors:** Danielle de Oliveira Nascimento, Adriana Vieira-de-Abreu, Angélica F. Arcanjo, Patricia Torres Bozza, Guy A. Zimmerman, Hugo Caire Castro-Faria-Neto

**Affiliations:** ^1^Laboratório de Immunofarmacologia, Instituto Oswaldo Cruz, Fundação Oswaldo Cruz, Rio de Janeiro, Brazil; ^2^Department of Internal Medicine, University of Utah School of Medicine, Salt Lake City, UT, United States

**Keywords:** *Salmonella enterica* serovar Typhimurium pathogenesis, non-typhoidal *Salmonella*, myeloid leukocytes, macrophage, integrin, pyroptosis

## Abstract

β_2_ integrins are critical in host defense responses to invading pathogens and inflammation. Previously, we reported that genetic deficiency of integrin α_D_β_2_ in mice altered outcomes in experimental systemic infections including accelerated mortality in animals infected with *Salmonella enterica* serovar Typhimurium. Here, we show that deficiency of α_D_β_2_ results in impaired accumulation of leukocytes in response to peritoneal infection by *S*. Typhimurium, impaired pathogen clearance *in vivo*, defective bacterial elimination by cultured peritoneal macrophages, and enhanced pyroptosis, a cell death process triggered by *Salmonella*. *Salmonella*-infected animals deficient in α_D_β_2_ had increased levels of peritoneal cytokines in addition to other markers of pyroptosis, which may contribute to inflammatory injury and increased mortality in the context of impaired bacterial killing. These observations indicate important contributions of leukocyte integrins to the host response in experimental *Salmonella* infection and reveal previous activities of α_D_β_2_ in bacterial infection.

## Introduction

Trafficking of leukocytes in tissues and organs, and cell–cell interactions between immune effector cells, are essential for defense against pathogens, innate and acquired immunity, and physiologic and pathologic inflammation (1, 2). Integrins are critical in these processes. Integrins are transmembrane heterodimers that are broadly expressed in varying patterns on metazoan cells (3, 4). In addition to cellular trafficking, integrins mediate adhesion-dependent cellular localization, signaling, intercellular communication, interactions with matrix, and regulation of apoptosis (3, 5). A subfamily of integrins called the β_2_ or CD11/CD18 integrins, also termed leukocyte integrins or leukointegrins, consists of four members: α_L_β_2_ (6), α_M_β_2_ (CD11b/CD18, MAC-1, CR3), α_X_β_2_ (CD11c/CD18), and α_D_β_2_ (CD11d/CD18) (3, 7). As in all integrins, the α subunits are not expressed on the cell surface without non-covalent heterodimerization with the β subunit, and *vice versa* (3–5). Leukocyte integrin heterodimers are expressed in varying patterns on circulating myeloid leukocytes and lymphocytes and on tissue leukocytes, including macrophages and dendritic cells, and have important activities in infection, inflammation, and immunity (8–10). Critical roles of leukocyte integrins in defense against bacterial infection, innate immune responses, wound surveillance, and tissue repair are clearly demonstrated by heritable human leukocyte adhesion deficiency syndromes in which the expression of β_2_ integrins is absent or dramatically reduced, or inside-out signaling of integrin heterodimers is impaired (10). Experimental models in which β_2_ integrins are genetically deleted or blocked with specific antibodies confirm essential physiologic contributions of leukocyte integrins in host defense and inflammation (5, 7–10). Additional integrins of the β_1_ and other integrin subclasses are also found on individual leukocyte types depending on their lineage and activation state (5, 7, 9, 10).

The most recently discovered member of the leukocyte, or β_2_, integrin subfamily is α_D_β_2_ (CD11d/CD18), which is expressed on circulating leukocytes and tissue macrophages in humans and on a subpopulation of blood leukocytes and distinct subsets of macrophages in uninfected mice (6, 10–14). Experiments with primary leukocytes from humans or mice or with transfected cell lines indicate that α_D_β_2_ recognizes ICAM-3, ICAM-1, VCAM-1, and various matrix proteins (6, 11, 13–15). *In vivo* studies of wild type (WT) and $\alpha_{\text{D}}^{\text{–/–}}$ demonstrate that α_D_β_2_ expression can be dynamically modulated in response to infection and that it has complex activities in immune regulation and systemic inflammatory responses (12, 13). Nevertheless, little is known about the functions of α_D_β_2_ in infectious and inflammatory syndromes (10).

We previously reported that α_D_β_2_ expression is altered on splenic and hepatic macrophages in response to the rodent malarial pathogen, *Plasmodium berghei* Anka, and that targeted deletion of α_D_ conferred a survival advantage in this model of severe malaria (13). In parallel, we examined mice infected with *Salmonella enterica* serovar Typhimurium (16) to determine if genetic deletion of α_D_β_2_ has stereotyped or, conversely, differential effects in lethal systemic infections. *S. enterica* is an intracellular facultative anaerobe that is one of the leading causes of enteric diseases in the United States. *Salmonella* can cause diseases such as typhoid, gastroenteritis, bacteremia, and chronic asymptomatic carriage (17). *S. enterica* serovar Typhimurium, the causative agent of gastroenteritis, has developed different strategies to evade host defense mechanisms (18). In contrast to increased survival at early time points in the experimental malaria model, mortality was accelerated in $\alpha_{\text{D}}^{\text{–/–}}$ infected with *S*. Typhimurium (13). Here, we further explore functions of α_D_β_2_ in animals challenged with *S*. Typhimurium.

## Materials and Methods

### Reagents

Lipopolysaccharide (LPS) from *Escherichia coli* (serotype 0127:b8) and from *S. enterica* serotype Typhimurium were purchased from Sigma-Aldrich. Live/Dead BacLight bacterial viability kit and latex beads were obtained from Molecular Probes. Alexa 488-conjugated mAb 205c (13) was used to label α_D_ for flow cytometry analysis. Anti-TLR4-PE was purchased from eBioscience, San Diego, CA, USA. YVAD (N-Ac–Tyr–Val–Ala–Asp–CMK), a caspase-1 inhibitor, was purchased from Cayman-Chemical. Fluorochrome inhibitor of caspase (FLICA) was purchased from Immunochemistry Technologies (Bloomington, MN, USA). Necrostatin-1 (Nec-1) was obtained from Sigma-Aldrich (Taufkirchen, Germany). Nec-1 is an inhibitor of the receptor interacting protein 1 kinase and acts as an inhibitor of necroptosis (19).

### Mice

C57Bl/6 WT mice and mice with targeted deletion of α_D_ $\left(\alpha_{\text{D}}^{\text{–/–}}\right)$ to yield deficiency of α_D_β_2_ weighing 20–25 g of both sexes were obtained from the Oswaldo Cruz Foundation breeding unit. We have previously characterized these $\alpha_{\text{D}}^{\text{–/–}}$ mice in detail. The animals were kept at constant temperature (25^∘^C) with free access to food and water in a room with a 12-h light/dark cycle. The protocols employed in this work were approved by the Oswaldo Cruz Foundation Animal Welfare Committee under the license number 0011-00.

### Bacterial Strains

*Salmonella enterica* serovar Typhimurium (16) ISC 5302/2004 was a generous gift from the Department of Microbiology of the Oswaldo Cruz Foundation. The bacteria were cultured in Luria-Bertani broth (Guria Broth Miller; Sigma-Aldrich) for 16–18 h at 37^∘^C to obtain stationary growth phase cultures and were then centrifuged (1,000 × *g*) for 10 min at 4^∘^C. The pellets were resuspended in PBS to an OD of 0.1 at 660 nm, corresponding to 10^8^ CFU/mL. WT and $\alpha_{\text{D}}^{\text{–/–}}$ mice were infected by intraperitoneal (i.p.) injection of 200 μL of the bacteria suspension (10^5^ CFU *Salmonella*/mouse). Control WT mice were sham injected (saline alone) in parallel.

As YVAD (N-Ac–Tyr–Val–Ala–Asp–CMK) is a selective and irreversible inhibitor of interleukin-1 beta converting enzyme (Caspase-1), the effect of caspase-1 action on the absence of $\alpha_{\text{D}}^{\text{–/–}}$ during *Salmonella* Typhimurium infection was analyzed *in vivo*. For this, in some experiments, 15 min after *S*. Typhimurium infection, WT and $\alpha_{\text{D}}^{\text{–/–}}$ mice were injected i.p. with YVAD (200 μg/animal) or Nec-1 (1.65 mg Nec-1/kg body weight). Animals injected with sterile saline or DMSO served as controls.

### Leukocytes Counts

Total leukocyte counts were performed in Neubauer chambers by means of an optical microscope after diluting the peritoneal wash in Turk’s solution (2% acetic acid). Differential leukocyte analysis was performed under an oil immersion objective on cytocentrifuged cells stained with May-Grunwald–Giemsa dye.

### Cytokine Measurements

Levels of IL-6, TNF-α, MIP-1α, and IL-1β in peritoneal fluid were evaluated by enzyme-linked immunosorbent assay measurements using specific monoclonal antibodies, according to manufacturer instructions (Duo Set Kit from R&D Systems). Mice were sacrificed in a CO_2_ chamber at designated time points and the peritoneal cavities were opened and rinsed with PBS. The particulate matter was removed by centrifugation (252 × *g*) for 10 min, and the supernatant fractions were used for immunoassays.

### Intraperitoneal LPS Challenge

The administration of LPS, an endotoxin present on the cell wall of Gram-negative bacteria, has been extensively used in the study of sepsis, since it promotes the development of clinical signs observed in patients with this syndrome (20). Thus, as *S*. Typhimurium is a Gram-negative bacteria, we have determined whether LPS inoculation would alter the response pattern in $\alpha_{\text{D}}^{\text{–/–}}$ mice. For this, mice were injected intraperitoneally with 500 ng per cavity of LPS diluted in sterile saline. Control animals received equal volumes of saline.

### Toll-Like Receptor 4 (TLR4) Molecule Labeling

Toll-like receptor 4 is an important PRR receptor (pattern recognition receptor) for Gram-negative bacteria recognition. We use TLR4 molecule labeling for investigation of a possible activation pathway modulated by α_D_β_2_ integrin. TLR4 molecule from peritoneal cells of WT and $\alpha_{\text{D}}^{\text{–/–}}$ mice was labeled by Flow cytometry analysis, according to manufacturer instructions (eBioscience, San Diego, CA, USA).

### Measurement of Caspase-1 Activity

Caspase-1 activity was measured with FAM-YVAD FMK (5-carboxyfluorescein–Tyr–Val–Ala–Asp–fluoromethylketone, FLICA) in peritoneal cells of WT and $\alpha_{\text{D}}^{\text{–/–}}$ mice, infected or not. The addition of FLICA was done according to the manufacturer’s instructions (Immunochemistry Technologies, Bloomington, MN, USA). FLICA^+^ cells were detected by FACS analysis.

### Macrophage Culture and *In Vitro* Infection

Peritoneal cells from WT (16) C57Bl/6 and $\alpha_{\text{D}}^{\text{–/–}}$ mice were collected by lavage with sterile RPMI 1640 cell culture medium. Macrophages (1 × 10^6^ cells/mL) were allowed to adhere for 2 h at 37^∘^C in a 5% CO_2_ atmosphere with RPMI 1640 cell culture medium containing 2% FCS. The non-adherent cells were removed after vigorous PBS wash. Macrophages were incubated with *S*. Typhimurium (10^5^ CFU/well) (21) or with fluorescent beads (FluoSpheres® polystyrene microspheres), 1.0 μm, yellow-green fluorescent (Invitrogen), or IDC Surfactant-free Latex Beads (Invitrogen) (1:1), for 1 h at 37^∘^C in 5% CO_2_ atmosphere. After this incubation period, the cultures were washed three times with PBS to remove non-internalized bacteria or beads. Then, the cells were incubated in RPMI 1640 cell culture medium for 1 h at 37^∘^C in a 5% CO_2_ atmosphere in the absence or presence of 5 μg/mL gentamicin to kill the remaining extracellular bacteria. Sterile saline was used as control. After incubation, macrophages were lysed with 0.1% saponin for 20 min at room temperature. Aliquots of the cell lysates were plated on tryptic soy agar medium and incubated at 37^∘^C for CFU determinations. The cell-free supernatants were recovered and stored at −20^∘^C.

### Analysis of Nitric Oxide

Analysis of the presence of nitric oxide was performed using the DAF-FM probe (D-23842, Molecular Probes), according to manufacturer instructions. As the maximum length of excitation and emission fluorescence are 495 and 515 nm, respectively, and therefore similar to fluorescein (FITC), a flow cytometer was used.

### Flow Cytometry

After adjusting the concentration of cell samples to 1–2 × 10^6^ cells/100 μL specific antibodies and their respective control antibodies were incubated with the leukocytes for 30 min on ice. Samples were washed with FACS medium (HBSS without Ca^2+^ and Mg^2+^/0.1% NaN_3_/0.5% human serum albumin) and fixed in 3.7% formalin. The samples were stored at 4^∘^C and protected from light until analysis.

For cell size analysis, we utilized a single parameter histogram along the *X*-axis (FCS—Forware Scatter), gating on the peritoneal macrophage region. This parameter is a measurement of the amount of the laser beam that passes around the cell, yielding a relative size for the cell. Cells were analyzed by flow cytometry using a FACSCalibur® device (Becton Dickinson, San Jose, CA, USA) equipped with CellQuest software. A gate excluding cell debris and non-viable cells was utilized. Analyses were done after recording 10,000 events for each sample.

### Statistical Analysis

All data are expressed as mean ± SEM and compared using a two-tailed Student’s *t*-test and one-way ANOVA. Data were considered statistically significant with a *p* ≤ 0.05.

## Results

### Cellular Host Defense Responses to *S*. Typhimurium Are Differentially Altered in Mice Deficient in ***α***_D_***β***_2_

**Figure 1:**
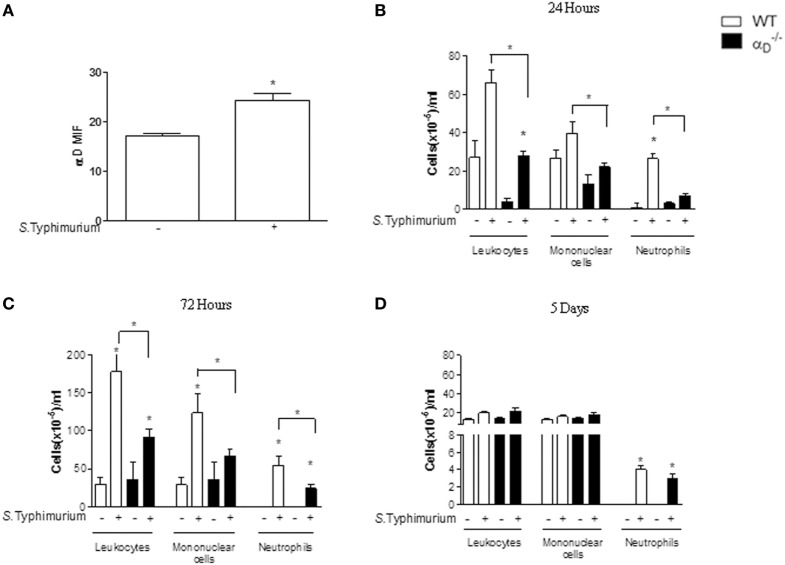
Integrin α_D_β_2_ influences leukocyte accumulation in the peritoneal cavities of mice infected with *Salmonella* Typhimurium. Wild-type (WT) and $\alpha_{\text{D}}^{\text{–/–}}$ mice were infected with *S*. Typhimurium by intraperitoneal injection (10^5^ CFU/animal). Control animals were sham-infected by intraperitoneal injection of sterile, apyrogenic saline. **(A)** After 72 h of infection, we collected leukocytes by peritoneal lavage for analysis of α_D_β_2_ expression by flow cytometry [mean fluorescence intensity (MIF)]. In addition, the numbers of total leukocytes, mononuclear cells, and neutrophils in peritoneal fluid samples were determined at **(B)** 24 h, **(C)** 72 h, or **(D)** 5 days after infection. Leukocytes, mononuclear cells, and neutrophils differentiation was performed under an oil immersion objective on cytocentrifuged cells stained with May-Grunwald–Giemsa dye. Each bar indicates the mean ± SEM from five animals. The figures are representative of the results in four separate experiments. Individual asterisks indicate significant differences (*p* < 0.05) between infected and control animals. Bars with asterisks indicate significant differences between $\alpha_{\text{D}}^{\text{–/–}}$ and WT animals.

We utilized an i.p. challenge model of *Salmonella* infection to mimic intraperitoneal and systemic infectious complications, which occur clinically (22), and to relate the findings to previous observations (13, 23). Infection of WT C57BL/6 mice with *S*. Typhimurium increased expression of α_D_β_2_ on peritoneal macrophages when analyzed by flow cytometry (Figure 1A; Figure S1 in Supplementary Material), indicating that this integrin is involved in the host response to *Salmonella*. Because myeloid leukocyte accumulation is a feature of *S*. Typhimurium infection (23, 24), we examined inflammatory cell numbers in WT and $\alpha_{\text{D}}^{\text{–/–}}$ mice subjected to i.p. challenge with this pathogen. There was impaired leukocyte accumulation in the peritoneal cavities of $\alpha_{\text{D}}^{\text{–/–}}$ mice mainly with 72 h after infection, with substantially decreased numbers of total leukocytes, mononuclear cells, and neutrophils recovered in peritoneal fluid when compared with WT-infected animals (Figure 1C). The cause of the difference in cellularity observed in Figure 1B is unknown, but it is possible that it is related to the intrinsic conditions of the groups tested. This result demonstrates that α_D_β_2_ influences acute inflammatory cellular responses to intraperitoneal infection by *Salmonella*. After 5 days of infection, however, there were no significant differences in the numbers of mononuclear leukocytes or neutrophils or in total leukocyte numbers in peritoneal fluid samples from WT or $\alpha_{\text{D}}^{\text{–/–}}$ animals (Figure 1D). This indicates that alternative mechanisms, likely involving other β_2_ integrins and/or other members of the integrin family expressed by leukocytes (7, 9, 10), contribute to peritoneal leukocyte accumulation at later time points. Consistent with this, we found that α_M_ was increased on peritoneal leukocytes from WT and $\alpha_{\text{D}}^{\text{–/–}}$ animals at 72 h after i.p. infection when examined by flow cytometric analysis, indicating increased expression of α_M_β_2_ (Figure S2 in Supplementary Material).

**Figure 2:**
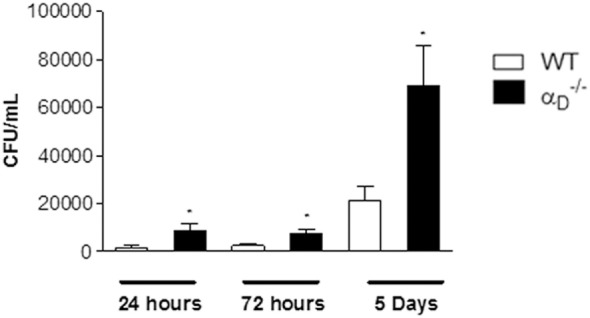
Targeted deletion of integrin α_D_β_2_ leads to a defect in peritoneal killing of *Salmonella* Typhimurium. Wild-type (WT) and $\alpha_{\text{D}}^{\text{–/–}}$ mice were infected with *S*. Typhimurium (10^5^ CFU/animal) by intraperitoneal injection or were sham-infected with sterile, apyrogenic saline as in Figure 1. Peritoneal fluid was collected 24 h, 72 h, or 5 days later, and the number of colony-forming units was determined in each sample. Each bar indicates the mean ± SEM of determinations in samples from five animals. The data in this figure are representative of three separate experiments. Significant differences (*p* < 0.05) between infected animals and controls are indicated by asterisks.

We also determined if genetic deficiency of α_D_β_2_ influences bacterial clearance in this model. The number of colony-forming units per milliliter (CFU/mL) of *Salmonella* in peritoneal fluid (Figure 2), in spleen and blood (Figure S3 in Supplementary Material) was higher in $\alpha_{\text{D}}^{\text{–/–}}$ mice compared with WT animals at 24 h, 72 h, and 5 days of infection. Together with the analysis of cell numbers (Figure 1), these findings indicate that α_D_β_2_ mediates leukocyte responses and mechanisms involved in clearance and/or multiplication of *Salmonella* in the infected peritoneal cavity.

**Figure 3:**
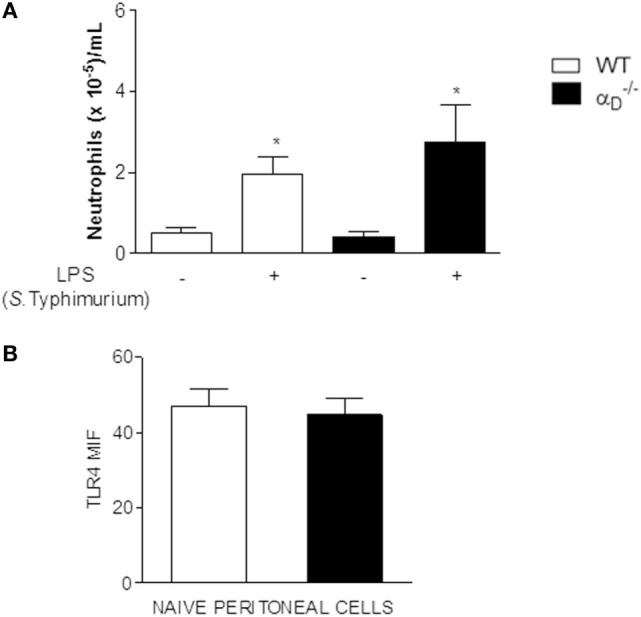
Recognition of *Salmonella* Typhimurium lipopolysaccharide (LPS), LPS signaling, and toll-like receptor 4 (TLR4) expression are not altered in $\alpha_{\text{D}}^{\text{–/–}}$ mice. **(A)** $\alpha_{\text{D}}^{\text{–/–}}$ and wild-type (WT) mice were challenged with *S*. Typhimurium LPS (500 ng) by intraperitoneal injection or injected with sterile, apyrogenic saline as a control. Peritoneal fluid was collected 24 h later, and the numbers of leukocytes determined. Asterisks indicate significant differences between LPS- and saline-injected animals. **(B)** Peritoneal cells were collected from $\alpha_{\text{D}}^{\text{–/–}}$ and WT animals in the absence of bacterial infection or LPS challenge and analyzed for TLR4 expression by flow cytometry [mean fluorescence intensity (MIF)]. Each bar indicates the determinations in samples from ≥4 animals. The data in this figure are representative of three separate experiments.

While β_2_ integrins have been reported to participate in *S*. Typhimurium recognition and dissemination based on knockout of all β_2_ (CD18) heterodimers (25), little is known about the contributions of individual leukocyte integrins to host defense against this bacterium and to the pathogenesis of *Salmonella* infections. Therefore, we examined other features of *Salmonella* challenge in $\alpha_{\text{D}}^{\text{–/–}}$ mice. We first excluded the possibility that TLR4 expression and recognition of *Salmonella* LPS are altered in $\alpha_{\text{D}}^{\text{–/–}}$. There were no differences in peritoneal neutrophil accumulation in WT and $\alpha_{\text{D}}^{\text{–/–}}$ mice 24 h after instillation of *S*. Typhimurium LPS (Figure 3A), although the cell numbers in both WT and $\alpha_{\text{D}}^{\text{–/–}}$ animals were lower than in mice challenged with live *Salmonella* bacteria (Figure 1). Consistent with intact recognition of LPS, the expression of TLR4 was not different on leukocytes from WT and $\alpha_{\text{D}}^{\text{–/–}}$ animals (Figure 3B).

### The Cytokine Response Is Altered in $\alpha {\;}_{\text{D}}^{\text{–/–}}$ Mice Infected With *Salmonella*

**Figure 4:**
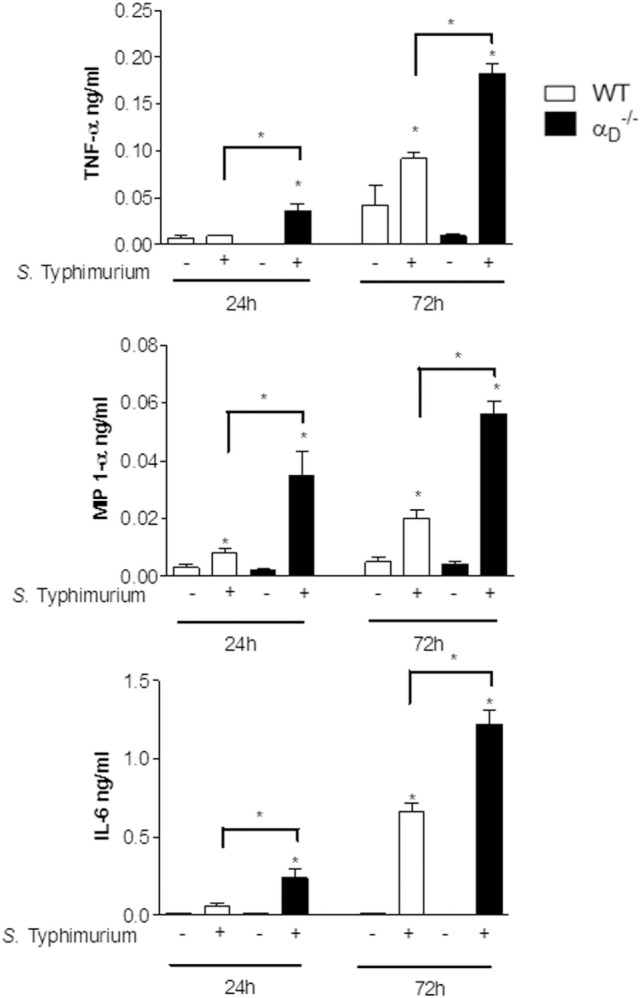
Inflammatory cytokines are increased in the peritoneal fluid of $\alpha_{\text{D}}^{\text{–/–}}$ mice infected with *Salmonella* Typhimurium. Wild-type (WT) and $\alpha_{\text{D}}^{\text{–/–}}$ mice were infected with *S*. Typhimurium (10^5^ CFU/animal) or sham-infected with sterile saline as in Figure 1. Peritoneal fluid was collected 24 or 72 h after challenge, and concentrations of TNF-α, MIP-1α, and IL-6 were determined. Each bar indicates the mean ± SEM of determinations in samples from five animals. Significant differences (*p* < 0.05) between infected animals and controls are indicated by bars and asterisks as in Figure 1. The data in this figure are representative of three separate experiments.

In parallel to analysis of peritoneal leukocyte accumulation in $\alpha_{\text{D}}^{\text{–/–}}$ mice challenged with *S*. Typhimurium, we also examined release of cytokines, which are central mediators in the host response to microbial pathogen invasion. Unexpectedly, there were higher levels of TNFα, MIP-1α, and IL-6 in the peritoneal fluid of infected $\alpha_{\text{D}}^{\text{–/–}}$ mice compared with levels in samples from WT controls (Figure 4). Thus, although total peritoneal leukocyte numbers and the numbers of myeloid leukocytes were decreased in $\alpha_{\text{D}}^{\text{–/–}}$ mice at early time points (Figure 1), α_D_β_2_-deficient animals had amplified peritoneal accumulation of pro-inflammatory cytokines.

### Integrin ***α***_D_***β***_2_ Modulates *Salmonella* Killing and Release of Cytokines by Peritoneal Macrophages *In Vitro*

**Figure 5:**
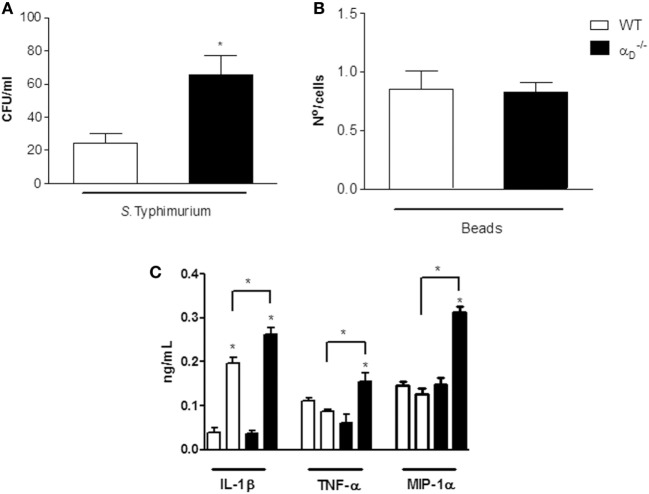
Integrin α_D_β_2_ regulates *Salmonella* Typhimurium killing and release of inflammatory cytokines by peritoneal macrophages challenged *in vitro*. **(A)** Peritoneal macrophages (10^6^ cells/mL) were collected from wild-type (WT) or $\alpha_{\text{D}}^{\text{–/–}}$ mice and challenged *in vitro* with *S*. Typhimurium (10^5^ CFU/mL) or incubated in medium alone for 1 h at 37^∘^C. The medium was removed, and the cells were incubated for an additional hour with fresh medium without antibiotics. After 1 h of incubation at 37^∘^C, the macrophages were lysed with saponin (0.1%), and bacterial CFU were determined in each sample. **(B)** WT and $\alpha_{\text{D}}^{\text{–/–}}$ peritoneal macrophages were incubated with latex particles (10^7^/well). The media were removed, and fresh media added as in panel **(A)**, and the number of intracellular beads counted by light microscopy. Each bar indicates the mean ± SEM of beads in 100 consecutively counted cells. **(C)** Supernatants were collected from peritoneal macrophages infected with *S*. Typhimurium or from uninfected peritoneal macrophages cultured as described in panel **(A)** after 1 h of incubation and assayed for IL-1β, TNF-α, and MIP-1α **(C)**. Three to five wells were analyzed in each experiment. The data in this figure are representative of three experiments with similar results. Significant differences (*p* < 0.05) between infected and control cells are indicated by asterisks and between cells from $\alpha_{\text{D}}^{\text{–/–}}$ and WT animals by bars with asterisks.

We then examined bacterial elimination and cytokine release by isolated peritoneal macrophages to mechanistically correlate these activities with *in vivo* determinations. We found that peritoneal macrophages from $\alpha_{\text{D}}^{\text{–/–}}$ mice cultured with *S*. Typhimurium *in vitro* contained increased numbers of viable bacteria compared with numbers of *Salmonella* in WT peritoneal macrophages (Figure 5A). Impaired elimination of *Salmonella* by $\alpha_{\text{D}}^{\text{–/–}}$ macrophages *in vitro* (Figure 5A) parallels defective *in vivo* elimination of the pathogen by $\alpha_{\text{D}}^{\text{–/–}}$ mice that we observed (Figure 2). Ingestion of microbeads by $\alpha_{\text{D}}^{\text{–/–}}$ and WT macrophages was similar (Figure 5B), indicating that global phagocytosis is intact in $\alpha_{\text{D}}^{\text{–/–}}$ macrophages and consistent with increased intracellular CFU in macrophages cultured with the pathogen (Figure 5A). In addition, we found that peritoneal macrophages from $\alpha_{\text{D}}^{\text{–/–}}$ mice released increased levels of IL-1β, TNFα, and MIP-1α compared with WT macrophages when challenged with *S*. Typhimurium *in vitro* (Figure 5C). This pattern was again similar to that in animals challenged with the bacterium *in vivo* (Figure 4) and indicates that peritoneal macrophages are a potential source of elevated cytokine levels in the peritoneal exudate in infected $\alpha_{\text{D}}^{\text{–/–}}$ mice.

### *Salmonella* Typhimurium Infection of $\alpha {\;}_{\text{D}}^{\text{–/–}}$ Mice Triggers Enhanced Pyroptosis

**Figure 6:**
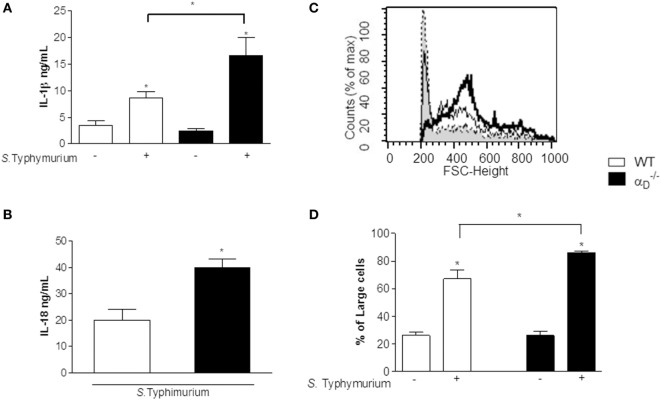
IL-1β, IL-18, and leukocyte swelling, key markers of pyroptosis, are increased in peritoneal fluid from *Salmonella-*infected α_D_β_2_-deficient mice. **(A,B)** Wild-type (WT) and $\alpha_{\text{D}}^{\text{–/–}}$ mice were infected with *Salmonella* Typhimurium (10^5^ CFU/animal) or were sham-infected as described in Figure 1. Peritoneal fluid was collected 72 h later, and concentrations of IL-1β **(A)** and IL-18 **(B)** were determined. Significant differences (*p* < 0.05) between infected and sham-infected mice are indicated by asterisks and between $\alpha_{\text{D}}^{\text{–/–}}$ and WT animals by a bar and asterisk. The data in panels **(A,B)** summarize the results of two experiments performed separately with samples from a minimum of three mice for each condition in each experiment. **(C)** Histograms indicating the size parameters of cells in peritoneal fluid from WT and $\alpha_{\text{D}}^{\text{–/–}}$ control and infected animals are shown: dashed line and gray shading, cells from control WT and $\alpha_{\text{D}}^{\text{–/–}}$ mice; thin black line, WT-infected mice; thick black line, $\alpha_{\text{D}}^{\text{–/–}}$-infected mice. The *Y* -axis indicates cell number, and the *X*-axis indicates fluorescence intensity (FSC). **(D)** The fraction of larger cells in samples from control and infected animals of each genotype is shown. Significant differences (*p* ≤ 0.005) between infected animals and controls are indicated by asterisks and between WT and $\alpha_{\text{D}}^{\text{–/–}}$ mice by a bar and asterisk. Each bar represents the mean ± SEM of determinations in samples from ≥4 animals. The data in this figure are representative of two experiments with similar results.

Pyroptosis is a host response to *Salmonella* that involves a cell death program and resultant release of pro-inflammatory cytokines by infected macrophages (26–30). Caspase-1 is the inflammatory caspase crucial to canonical inflammasome-mediated pyroptosis and cytokine maturation (31). Enhanced release of IL-1β and IL-18, resulting from caspase-1-mediated cleavage of the pro-forms of these inflammatory proteins to yield mature, bioactive cytokines, is a defining feature of pyroptosis although other cytokines including IL-6 and TNFα are also released in an enhanced fashion (28, 29). Therefore, we measured IL-1β and IL-18 in peritoneal fluid from mice infected 72 h previously with *S*. Typhimurium and found that these interleukins were increased in samples from $\alpha_{\text{D}}^{\text{–/–}}$ mice when compared with levels in peritoneal fluid from WT animals (Figures 6A,B). Thus, these key markers of pyroptosis are increased in the peritoneal inflammatory response of $\alpha_{\text{D}}^{\text{–/–}}$ mice infected by *Salmonella*.

**Figure 7:**
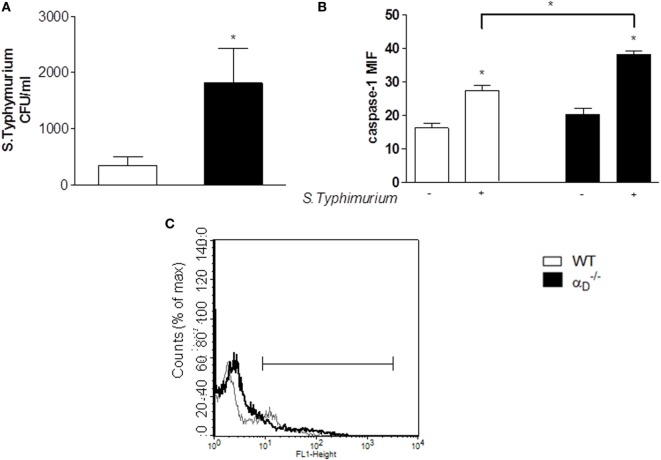
Caspase-1 is increased in peritoneal leukocytes from *Salmonella* Typhimurium-infected mice. **(A)** Mice were infected with *S*. Typhimurium as in Figure 1 or were sham-infected, and 24 h later peritoneal fluid was collected and cultured. There was increased growth of *S*. Typhimurium in the samples from $\alpha_{\text{D}}^{\text{–/–}}$ animals, similar to that in experiments shown in Figure 2. **(B,C)** Cells from the peritoneal fluid samples were also analyzed for active caspase-1 by flow cytometry as described in Sections “Materials and Methods” and “Results” [mean fluorescence industry (MIF)]. Each bar indicates the mean ± SEM of determinations in samples from ≥4 animals. Significant differences (*p* ≤ 0.05) between infected and sham-infected mice were indicated by asterisks, and between $\alpha_{\text{D}}^{\text{–/–}}$ and wild-type (WT) mice were indicated asterisks and bars. **(C)** Histogram indicating the active caspase-1 expression in peritoneal cells from WT- and $\alpha_{\text{D}}^{\text{–/–}}$-infected mice is shown: thin gray line represents cells from WT-infected mice, and thick black line represents cells from $\alpha_{\text{D}}^{\text{–/–}}$-infected mice. The *Y* -axis indicates cell number, and the *X*-axis indicates fluorescence intensity.

Analysis of peritoneal cells demonstrated increased cell size in samples from $\alpha_{\text{D}}^{\text{–/–}}$ animals compared with cells in the peritoneal fluids of WT mice after infection with *Salmonella* (Figures 6C,D). Cell swelling, eventual osmotic cell lysis, and release of cytokines are central features of pyroptosis (26, 28, 29). In parallel, we found increased caspase-1 activity in peritoneal macrophages from *S*. Typhimurium-infected $\alpha_{\text{D}}^{\text{–/–}}$ mice (Figures 7B,C) using a cell-permeant fluorescent probe that specifically binds to activated caspase-1 (16). Caspase-1 activation is a defining mechanism in pyroptosis and is upstream of cell swelling, cell lysis, and cytokine release (26, 28–30, 32). Preliminary results after administration of Nec-1 *in vivo* showed no change in the leukocyte migration pattern between infected WT and $\alpha_{\text{D}}^{\text{–/–}}$ mice, suggesting that there is no necroptosis in our model (Figure S4 in Supplementary Material). Thus, each of the key cellular and biochemical features of pyroptosis is enhanced in $\alpha_{\text{D}}^{\text{–/–}}$ mice infected with *S*. Typhimurium (Figures 4, 6, and 7). There was impaired killing of *Salmonella* by $\alpha_{\text{D}}^{\text{–/–}}$ macrophages with increased caspase-1 activity (Figure 7A), consistent with *in vitro* experiments (Figure 5A) and increased numbers of bacteria in the peritoneal fluid of infected $\alpha_{\text{D}}^{\text{–/–}}$ mice (Figure 2).

## Discussion

This study identifies three previously unrecognized features of the biology of integrin α_D_β_2_ directly relevant to infection and inflammation: this specialized heterodimer influences early leukocyte accumulation at extravascular sites in mice infected with a bacterial pathogen, regulates *in vivo* and *in vitro* elimination of *S*. Typhimurium, and indirectly or directly influences pyroptosis, a complex leukocyte response triggered by infection with *Salmonella* and other microbes. In previous studies, antibodies raised against α_D_ (3, 4, 6), altered accumulation of macrophages and neutrophils at sites of experimental spinal cord injury (33, 34) or in non-infectious peritonitis caused by thioglycollate (35). Here, we found that the accumulation of mononuclear cells and neutrophils was depressed in the peritoneal cavities of mice genetically deficient in α_D_β_2_ when the animals were infected by *S*. Typhimurium, although the impairment in leukocyte accumulation was not sustained at 5 days after infection (Figure 1). A similar early—but temporally limited—influence of α_D_β_2_ on leukocyte accumulation in spinal cord injury in rats was previously reported (34). Integrin α_D_β_2_ mediates adhesiveness of primary murine macrophages (13, 15), mouse cell lines (15), and human leukocyte subsets and cell lines (6, 11, 14), and is also reported to alter monocyte/macrophage migration *in vitro* (35). Expression of α_D_β_2_ is increased on peripheral blood monocytes after peritoneal inflammation in mice (35). Thus, the defect in early myeloid leukocyte accumulation in the peritoneal cavities of *Salmonella-*infected $\alpha_{\text{D}}^{\text{–/–}}$ mice is likely in part due to impaired adhesion and/or migration during inflammatory trafficking. The residual ability of myeloid leukocytes to accumulate in the inflamed peritoneal cavities of $\alpha_{\text{D}}^{\text{–/–}}$ mice (Figure 1) may be due to other β_2_ integrins such as α_M_β_2_, which also contribute to adhesion, migration, and pathogen recognition by myeloid leukocytes (7, 35, 36).

Previous studies indicate that impairment of myeloid leukocyte accumulation in mice challenged with *S*. Typhimurium may be sufficient to disrupt killing and clearance of the pathogen by the infected host (24). Thus, early impairment of leukocyte accumulation in the infected peritoneal cavity (Figure 1) might account for the faster demise of α_D_β_2_-deficient animals challenged with *Salmonella* that we saw in previous experiments (13). Nevertheless, we also found additional alterations in host responses of infected $\alpha_{\text{D}}^{\text{–/–}}$ mice that may alter the natural history of the infection and contribute to increased mortality. There were greater numbers of colony-forming *Salmonella* in peritoneal fluid samples from $\alpha_{\text{D}}^{\text{–/–}}$ animals. The increased pathogen burden was seen not only at early time points but also at 5 days, after peritoneal leukocyte numbers had equalized in $\alpha_{\text{D}}^{\text{–/–}}$ and WT mice (Figures 1 and 2). We observed a similar impairment in bacterial elimination in $\alpha_{\text{D}}^{\text{–/–}}$ macrophages challenged with bacteria *in vitro* (Figure 5), suggesting one or more defects in intracellular killing of the *Salmonella* pathogen. The impaired elimination of *Salmonella* by $\alpha_{\text{D}}^{\text{–/–}}$ macrophages in our experiments was not due to a generalized defect in phagocytosis (Figure 5), which is mediated by specific β_2_ integrins (8).

Because integrins, including members of the leukocyte integrin subfamily, mediate outside-in signaling in addition to cellular adhesion and surface binding of ligands and microorganisms (3–5, 7, 9, 10), α_D_β_2_ may signal to critical pathways that determine *Salmonella* killing versus intracellular survival (37). In preliminary experiments, we found intact production of nitric oxide by macrophages from $\alpha_{\text{D}}^{\text{–/–}}$ mice (Figure S5 in Supplementary Material), suggesting that this is not the molecular explanation for defective elimination. While it is clear that β_2_ integrins regulate multiple leukocyte functions (5, 8, 10, 36), specific activities of individual β_2_ heterodimers—such as α_D_β_2_—in pathogen recognition, killing, and clearance are largely unexplored.

We also found evidence for increased pyroptosis in $\alpha_{\text{D}}^{\text{–/–}}$ mice challenged with i.p. *Salmonella*. *Salmonella* infection of macrophages induces complex cellular responses (37), including autophagy (38) and pyroptosis (27–30). Pyroptosis, originally characterized as caspase-1-dependent death of *Shigella*- or *Salmonella*-infected macrophages, occurs *in vitro* and *in vivo* (29, 30, 39–41), and may also be triggered by other bacteria including *Yersinia*, *Pseudomonas*, *Bacillus anthracis*, and *Burkholderia* (28, 41). Pyroptosis is mechanistically distinct from other forms of programmed cell death (26, 28, 30). Despite intensive studies of inflammasomes, the regulating mechanisms of IL-1β/IL-18 production and pyroptosis after caspase-1 (canonical pathway) or caspase-11 (non-canonical pathway) activation are unknown. Recent work has shown the participation of Gasdermin D as a new component involved in these processes (42–45). As there were no differences in peritoneal neutrophil accumulation in WT and $\alpha_{\text{D}}^{\text{–/–}}$ mice after LPS instillation (Figure 3A), our data suggest that in our model there is the involvement of the canonical pathway of inflammasome activation. In the non-canonical inflammasome, the inflammatory protease, caspase-11 acts as a receptor for LPS that gains access to the cytosol (46).

Caspase-1 activation and inflammasome-mediated production of mature IL-1β and IL-18, cellular swelling due to plasma membrane permeabilization, and release of pro-inflammatory cytokines are defining and/or critical features of pyroptosis (28–30, 47). We found that each of these essential markers of pyroptosis is enhanced in $\alpha_{\text{D}}^{\text{–/–}}$ mice infected with *S*. Typhimurium (Figures 5–7). Pyroptotic cell death of macrophages may contribute to reduced total numbers of leukocytes in the peritoneal fluid of $\alpha_{\text{D}}^{\text{–/–}}$ animals early after *Salmonella* infection (Figure 1).

Enhancement of pyroptosis in $\alpha_{\text{D}}\beta_{\text{2}}^{\text{–/–}}$ mice was an unexpected finding. Integrin signaling regulates apoptosis in some cell types (5, 48) and can alter activities of caspases 3 and 8 (36, 49, 50). Nevertheless, β_2_ integrins are not known to influence macrophage death or pyroptosis, or to specifically signal to caspase-1 or to events downstream of caspase-1 in the pyroptosis pathway (27–29). Our observation that pyroptosis is enhanced in $\alpha_{\text{D}}^{\text{–/–}}$ mice may indicate that outside-in signaling by α_D_β_2_ regulates caspase-1 activation and/or downstream events in the pyroptosis pathway such as inflammasome assembly or processing of pro-IL-1β and pro-IL-18 (28, 51). This novel possibility and the Gasdermin D participation remains to be further explored. Alternatively, increased numbers of bacteria in $\alpha_{\text{D2}}^{\text{–/–}}$ mice (Figures 2, 5A, and 7A; Figure S1 in Supplementary Material) may drive pyroptosis in this model. Pyroptosis is thought to limit bacterial replication and to be protective in infection by *Salmonella* and, potentially, other intracellular pathogens (26, 28–30, 52). Consistent with this interpretation, caspase-1-deficient mice are more susceptible to infection and to death when challenged with *Salmonella* (26, 30, 53, 54). Here again, a potential involvement of Gasdermin D needs to be better studied in our model, since Kambara et al. (55) showed that Gasdermin D deficiency unexpectedly and paradoxically augments host defenses against extracellular *E. coli* by delaying neutrophil death and by the increase in the host bactericidal activity to *E. coli* infection.

Nevertheless, while enhanced pyroptosis is predicted to have a protective effect (28–30, 52), this component appears to be outweighed by parallel defects in *Salmonella* elimination, with mechanisms yet to be established, in $\alpha_{\text{D}}^{\text{–/–}}$ animals. In this context, the enhanced release of cytokines by infected macrophages (Figures 4–6)—a key feature of pyroptosis (26, 28, 29)—may contribute to systemic inflammatory injury and to accelerated mortality in $\alpha_{\text{D}}^{\text{–/–}}$ animals, as we reported previously (13). Of note, we should say that increased cytokine production in $\alpha_{\text{D}}^{\text{–/–}}$ mice infected with *S*. Typhimurium could also be a consequence of the increased bacteria burden observed in those animals. Excessive, unregulated generation of pro-inflammatory cytokines is a central pathophysiologic mechanism in sepsis and other lethal systemic infectious and non-infectious syndromes (56, 57).

The potential for α_D_β_2_ to deliver outside-in signals to pathways that regulate cytokine synthesis in multiple leukocyte types adds to the complexity of the phenotype of $\alpha_{\text{D}}^{\text{–/–}}$ animals infected by *S*. Typhimurium or other pathogens.

Our observations indicate previously unrecognized activities of integrin α_D_β_2_ and reveal additional contributions of leukocyte integrins to the host response to experimental *S*. Typhimurium challenge. Infection with *S*. Typhimurium and other non-typhoidal *Salmonella* strains are major causes of invasive infection and bacteremia in humans (22, 58, 59). Thus, our identification of previously unrecognized activities of integrin α_D_β_2_ in experimental *S*. Typhimurium infection may have clinical relevance and lead to new mechanistic insights in human disease. In addition, we have found evidence for differential activities of α_D_β_2_ in experimental infections by *Salmonella* (this study) and malarial parasites (13), indicating that this leukocyte integrin has complex roles in host defense against invading pathogens. Further studies of these features of integrin α_D_β_2_ in models of infection may yield mechanistic information with translational relevance.

## Ethics Statement

The animals were kept at constant temperature (25^∘^C) with free access to food and water in a room with a 12-h light/dark cycle. The protocols employed in this work were approved by the Oswaldo Cruz Foundation Animal Welfare Committee under the license number 0011-00.

## Author Contributions

Conceived and designed the experiments: DN, GZ, and HC-F-N. Performed the experiments: DN, AV-d-A, and AA. Analyzed the data: DN, HC-F-N, PB, and GZ. Contributed reagents/materials/analysis tools: HC-F-N, PB, and GZ. Wrote the paper: DN, GZ, and HC-F-N.

## Conflict of Interest Statement

The authors declare that the research was conŋducted in the absence of any commercial or financial relationships that could be construed as a potential conflict of interest.

## References

[1] von Andrian UH Mackay CR T-cell function and migration. Two sides of the same coin. N Engl J Med (2000) 343:1020–34. 10.1056/NEJM200010053431407 11018170

[2] Medzhitov R Origin and physiological roles of inflammation. Nature (2008) 454:428–35. 10.1038/nature07201 18650913

[3] Hynes RO Integrins: bidirectional, allosteric signaling machines. Cell (2002) 110:673–87. 10.1016/S0092-8674(02)00971-6 12297042

[4] Barczyk M Carracedo S Gullberg D. Integrins. Cell Tissue Res (2010) 339:269–80. 10.1007/s00441-009-0834-6 19693543 PMC2784866

[5] Lowell CA Mayadas TN. Overview: studying integrins in vivo. Methods Mol Biol (2012) 757:369–97. 10.1007/978-1-61779-166-6_22 21909923 PMC3248401

[6] Van der Vieren M Le Trong H Wood CL Moore PF St John T Staunton DE A novel leukointegrin, alpha d beta 2, binds preferentially to ICAM-3. Immunity (1995) 3:683–90. 10.1016/1074-7613(95)90058-6 8777714

[7] Harris ES McIntyre TM Prescott SM Zimmerman GA The leukocyte integrins. J Biol Chem (2000) 275:23409–12. 10.1074/jbc.R000004200 10801898

[8] Lee SH Corry DB Homing alone? CD18 in infectious and allergic disease. Trends Mol Med (2004) 10:258–62. 10.1016/j.molmed.2004.04.002 15177189

[9] Evans R Patzak I Svensson L De Filippo K Jones K McDowall A Integrins in immunity. J Cell Sci (2009) 122:215–25. 10.1242/jcs.019117 19118214

[10] Harris ES Weyrich AS Zimmerman GA. Lessons from rare maladies: leukocyte adhesion deficiency syndromes. Curr Opin Hematol (2013) 20:16–25. 10.1097/MOH.0b013e32835a0091 23207660 PMC3564641

[11] Grayson MH Van der Vieren M Sterbinsky SA Michael Gallatin W Hoffman PA Staunton DE alphadbeta2 integrin is expressed on human eosinophils and functions as an alternative ligand for vascular cell adhesion molecule 1 (VCAM-1). J Exp Med (1998) 188:2187–91. 10.1084/jem.188.11.2187 9841932 PMC2212388

[12] Wu H Rodgers JR Perrard XY Perrard JL Prince JE Abe Y Deficiency of CD11b or CD11d results in reduced staphylococcal enterotoxin-induced T cell response and T cell phenotypic changes. J Immunol (2004) 173:297–306. 10.4049/jimmunol.173.1.297 15210787

[13] Miyazaki Y Bunting M Stafforini DM Harris ES McIntyre TM Prescott SM Integrin alphaDbeta2 is dynamically expressed by inflamed macrophages and alters the natural history of lethal systemic infections. J Immunol (2008) 180:590–600. 10.4049/jimmunol.180.1.590 18097061 PMC2275910

[14] Miyazaki Y Vieira de Abreu A Harris ES Shah AM Weyrich AS Castro-Faria-Neto HC Integrin α_D_β_2_ (CD11d/CD18) is expressed by human circulating and tissue myeloid leukocytes and mediates inflammatory signaling. PLoS One (2014) 9:e112770 10.1371/journal.pone.0112770 25415295 PMC4240710

[15] Yakubenko VP Yadav SP Ugarova TP. Integrin alphaDbeta2, an adhesion receptor up-regulated on macrophage foam cells, exhibits multiligand-binding properties. Blood (2006) 107:1643–50. 10.1182/blood-2005-06-2509 16239428 PMC1367263

[16] Broz P Newton K Lamkanfi M Mariathasan S Dixit VM Monack DM. Redundant roles for inflammasome receptors NLRP3 and NLRC4 in host defense against *Salmonella*. J Exp Med (2010) 207:1745–55. 10.1084/jem.20100257 20603313 PMC2916133

[17] Vance RE Isberg RR Portnoy DA Patterns of pathogenesis: discrimination of pathogenic and nonpathogenic microbes by the innate immune system.Cell Host Microbe. (2009) 6:10–21. 19616762 10.1016/j.chom.2009.06.007 PMC2777727

[18] Broz P Ohlson MB Monack DM Innate immune response to *Salmonella* Typhimurium, a model enteric pathogen. Gut Microbes (2012) 3(2):62–70. 10.4161/gmic.19141 22198618 PMC3370950

[19] Degterev A Hitomi J Germscheid M Ch’en IL Korkina O Teng X Identification of RIP1 kinase as a specific cellular target of necrostatins. Nat Chem Biol (2008) 4:313–21. 10.1038/nchembio.83 18408713 PMC5434866

[20] Suffredini AF Fromm RE Parker MM Brenner M Kovacs JA Wesley RA Parrillo JE The cardiovascular response of normal humans to the administration of endotoxin. Engl J Med. (1989) 321:280–7. 10.1056/NEJM198908033210503 2664516

[21] Van der Velden AW Lindgren SW Worley MJ Heffron F. *Salmonella* pathogenicity island 1-independent induction of apoptosis in infected macrophages by *Salmonella enterica* serotype Typhimurium. Infect Immun (2000) 68:5702–9. 10.1128/IAI.68.10.5702-5709.2000 10992474 PMC101526

[22] Hohmann EL. Nontyphoidal salmonellosis. Clin Infect Dis (2001) 32:263–9. 10.1086/318457 11170916

[23] Conlan JW North RJ Listeria monocytogenes, but not *Salmonella* Typhimurium, elicits a CD18-independent mechanism of neutrophil extravasation into the murine peritoneal cavity. Infect Immun (1994) 62:2702–6. 7911783 10.1128/iai.62.7.2702-2706.1994 PMC302871

[24] Wick MJ. Innate immune control of *Salmonella enterica* serovar Typhimurium: mechanisms contributing to combating systemic *Salmonella* infection. J Innate Immun (2011) 3:543–9. 10.1159/000330771 21912097

[25] Vazquez-Torres A Jones-Carson J Bäumler AJ Falkow S Valdivia R Brown W Extraintestinal dissemination of *Salmonella* by CD18-expressing phagocytes. Nature (1999) 401:804–8. 10.1038/44593 10548107

[26] Fink SL Cookson BT. Pyroptosis and host cell death responses during *Salmonella* infection. Cell Microbiol (2007) 9:2562–70. 10.1111/j.1462-5822.2007.01036.x 17714514

[27] Fink SL Bergsbaken T Cookson BT. Anthrax lethal toxin and *Salmonella* elicit the common cell death pathway of caspase-1-dependent pyroptosis via distinct mechanisms. Proc Natl Acad Sci U S A (2008) 105:4312–7. 10.1073/pnas.0707370105 18337499 PMC2393760

[28] Bergsbaken T Fink SL Cookson BT. Pyroptosis: host cell death and inflammation. Nat Rev Microbiol (2009) 7:99–109. 10.1038/nrmicro2070 19148178 PMC2910423

[29] Kepp O Galluzzi L Zitvogel L Kroemer G Pyroptosis – a cell death modality of its kind? Eur J Immunol (2010) 40:627–30. 10.1002/eji.200940160 20201017

[30] Miao EA Rajan JV Aderem A. Caspase-1-induced pyroptotic cell death. Immunol Rev (2011) 243:206–14. 10.1111/j.1600-065X.2011.01044.x 21884178 PMC3609431

[31] Lamkanfi M Dixit VM. Inflammasomes and their roles in health and disease. Annu Rev Cell Dev Biol (2012) 28:137–61. 10.1146/annurev-cellbio-101011-155745 22974247

[32] Yazdi AS Guarda G D’Ombrain MC Drexler SK. Inflammatory caspases in innate immunity and inflammation. J Innate Immun (2010) 2:228–37. 10.1159/000283688 20375549

[33] Mabon PJ Weaver LC Dekaban GA. Inhibition of monocyte/macrophage migration to a spinal cord injury site by an antibody to the integrin alphaD: a potential new anti-inflammatory treatment. Exp Neurol (2000) 166:52–64. 10.1006/exnr.2000.7488 11031083

[34] Saville LR Pospisil CH Mawhinney LA Bao F Simedrea FC Peters AA A monoclonal antibody to CD11d reduces the inflammatory infiltrate into the injured spinal cord: a potential neuroprotective treatment. J Neuroimmunol (2004) 156:42–57. 10.1016/j.jneuroim.2004.07.002 15465595

[35] Yakubenko VP Belevych N Mishchuk D Schurin A Lam SC Ugarova TP. The role of integrin alpha D beta2 (CD11d/CD18) in monocyte/macrophage migration. Exp Cell Res (2008) 314:2569–78. 10.1016/j.yexcr.2008.05.016 18621369 PMC2621015

[36] Mayadas TN Cullere X. Neutrophil beta2 integrins: moderators of life or death decisions. Trends Immunol (2005) 26:388–95. 10.1016/j.it.2005.05.002 15922663

[37] Haraga A Ohlson MB Miller SI. Salmonellae interplay with host cells. Nat Rev Microbiol (2008) 6:53–66. 10.1038/nrmicro1788 18026123

[38] Hernandez LD Pypaert M Flavell RA Galán JE. A *Salmonella* protein causes macrophage cell death by inducing autophagy. J Cell Biol (2003) 163:1123–31. 10.1083/jcb.200309161 14662750 PMC2173598

[39] Hersh D Monack DM Smith MR Ghori N Falkow S Zychlinsky A. The *Salmonella* invasin SipB induces macrophage apoptosis by binding to caspase-1. Proc Natl Acad Sci U S A (1999) 96:2396–401. 10.1073/pnas.96.5.2396 10051653 PMC26795

[40] Monack DM Hersh D Ghori N Bouley D Zychlinsky A Falkow S. *Salmonella* exploits caspase-1 to colonize Peyer’s patches in a murine typhoid model. J Exp Med (2000) 192:249–58. 10.1084/jem.192.2.249 10899911 PMC2193260

[41] Bergsbaken T Cookson BT. Macrophage activation redirects yersinia-infected host cell death from apoptosis to caspase-1-dependent pyroptosis. PLoS Pathog (2007) 3:e161. 10.1371/journal.ppat.0030161 17983266 PMC2048529

[42] Ding J Wang K Liu W She Y Sun Q Shi J Pore-forming activity and structural autoinhibition of the gasdermin family. Nature (2016) 535:111–6. 10.1038/nature18590 27281216

[43] He W-T Wan H Hu L Chen P Wang X Huang Z Gasdermin D is an executor of pyroptosis and required for interleukin-1b secretion. Cell Res (2015) 25:1285–98. 10.1038/cr.2015.139 26611636 PMC4670995

[44] Saeki N Usui T Aoyagi K Kim DH Sato M Mabuchi T Distinctive expression and function of four GSDM family genes (GSDMA-D) in normal and malignant upper gastrointestinal epithelium. Genes Chromosomes Cancer (2009) 48:261–71. 10.1002/gcc.20636 19051310

[45] Liu X Zhang Z Ruan J Pan Y Magupalli VG Wu H Inflammasome-activated gasdermin D causes pyroptosis by forming membrane pores. Nature (2016) 535:153–8. 10.1038/nature18629 27383986 PMC5539988

[46] Shi J Zhao Y Wang Y Gao W Ding J Li P Inflammatory caspases are innate immune receptors for intracellular LPS. Nature (2014) 514:187–92. 10.1038/nature13683 25119034

[47] Fink SL Cookson BT. Caspase-1-dependent pore formation during pyroptosis leads to osmotic lysis of infected host macrophages. Cell Microbiol (2006) 8:1812–25. 10.1111/j.1462-5822.2006.00751.x 16824040

[48] Frisch SM Ruoslahti E. Integrins and anoikis. Curr Opin Cell Biol (1997) 9:701–6. 10.1016/S0955-0674(97)80124-X 9330874

[49] Buckley CD Pilling D Henriquez NV Parsonage G Threlfall K Scheel-Toellner D RGD peptides induce apoptosis by direct caspase-3 activation. Nature (1999) 397:534–9. 10.1038/17409 10028971

[50] Stupack DG Puente XS Boutsaboualoy S Storgard CM Cheresh DA. Apoptosis of adherent cells by recruitment of caspase-8 to unligated integrins. J Cell Biol (2001) 155:459–70. 10.1083/jcb.200106070 11684710 PMC2150834

[51] Strowig T Henao-Mejia J Elinav E Flavell R. Inflammasomes in health and disease. Nature (2012) 481:278–86. 10.1038/nature10759 22258606

[52] Miao EA Leaf IA Treuting PM Mao DP Dors M Sarkar A Caspase-1-induced pyroptosis is an innate immune effector mechanism against intracellular bacteria. Nat Immunol (2010) 11:1136–42. 10.1038/ni.1960 21057511 PMC3058225

[53] Lara-Tejero M Sutterwala FS Ogura Y Grant EP Bertin J Coyle AJ Role of the caspase-1 inflammasome in *Salmonella* Typhimurium pathogenesis. J Exp Med (2006) 203:1407–12. 10.1084/jem.20060206 16717117 PMC2118315

[54] Raupach B Peuschel SK Monack DM Zychlinsky A. Caspase-1-mediated activation of interleukin-1beta (IL-1beta) and IL-18 contributes to innate immune defenses against *Salmonella enterica* serovar Typhimurium infection. Infect Immun (2006) 74:4922–6. 10.1128/IAI.00417-06 16861683 PMC1539628

[55] Kambara H Liu F Zhang X Liu P Bajrami B Teng Y Exerts anti-inflammatory effects by promoting neutrophil death. Cell Rep (2018) 22:2924–36. 10.1016/j.celrep.2018.02.067 29539421 PMC5878047

[56] Rittirsch D Flierl MA Ward PA. Harmful molecular mechanisms in sepsis. Nat Rev Immunol (2008) 8:776–87. 10.1038/nri2402 18802444 PMC2786961

[57] Behrens EM Canna SW Slade K Rao S Kreiger PA Paessler M Repeated TLR9 stimulation results in macrophage activation syndrome-like disease in mice. J Clin Invest (2011) 121:2264–77. 10.1172/JCI43157 21576823 PMC3104738

[58] MacLennan CA Gondwe EN Msefula CL Kingsley RA Thomson NR White SA The neglected role of antibody in protection against bacteremia caused by nontyphoidal strains of *Salmonella* in African children. J Clin Invest (2008) 118:1553–62. 10.1172/JCI33998 18357343 PMC2268878

[59] MacLennan CA Host defense against malaria favors *Salmonella*. Nat Med (2012) 18:21–2. 10.1038/nm.2636 22227659

