# Supplementary material for: Integrin αDβ2 (CD11d/CD18) Modulates Leukocyte Accumulation, Pathogen Clearance, and Pyroptosis in Experimental Salmonella Typhimurium Infection
Source: Front Immunol. 2018 May 24;9:1128. doi: 10.3389/fimmu.2018.01128 (PMC5977906; doi:10.3389/fimmu.2018.01128)
Supplement: Supplementary file 1 [file image_1.pdf]

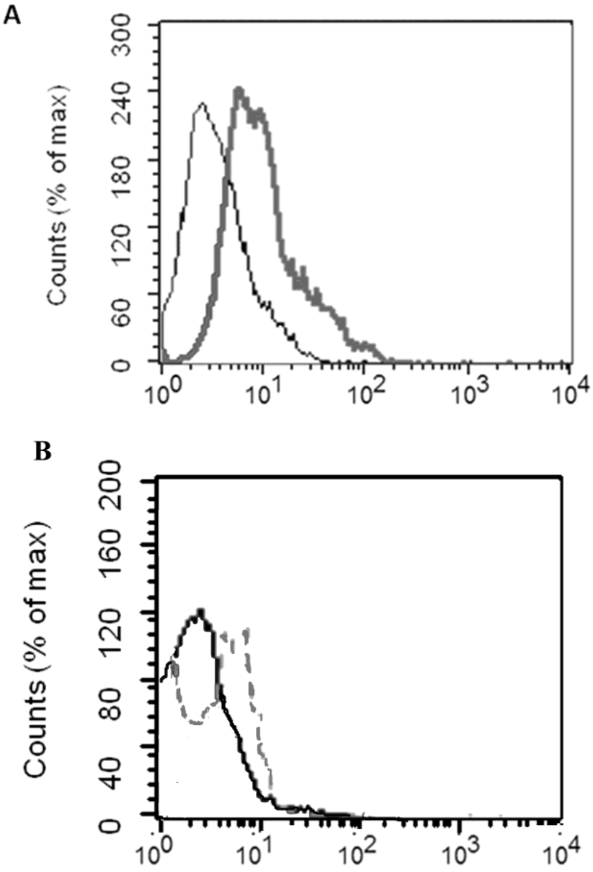

SUPPLEMENTARY FIGURE 1

Figure 1 - $\alpha_D\beta_2$ expression increased in wild type (WT) infected mice and it did not appear in infected $\alpha_D\beta_2^-$deficient mice.

Wild type (WT) and $\alpha_D^{-/-}$ mice were infected with *S.* Typhimurium by intraperitoneal injection (10^5 CFU/animal). Control animals were sham-infected by intraperitoneal injection of sterile, apyrogenic saline. After 72h of infection, we collected leukocytes by peritoneal lavage for analysis of $\alpha_D\beta_2$ expression by flow cytometry, as described in "Materials in Methods" (MIF, mean fluorescence intensity). Histograms indicating the $\alpha_D\beta_2$ expression in peritoneal cells from (A) Control and infected WT animals and (B) control and infected $\alpha_D^{-/-}$ animals are shown. (A) Thin black line represents cells from control WT and thick gray line cells from WT infected mice. (B) thick black line represents cell from $\alpha_D^{-/-}$ control mice and

dashed gray line cells from $\alpha_D^{-/-}$ infected mice. The Y axis indicates cell number and the X axis indicates fluorescence intensity.
