# Supplementary material for: Integrin αDβ2 (CD11d/CD18) Modulates Leukocyte Accumulation, Pathogen Clearance, and Pyroptosis in Experimental Salmonella Typhimurium Infection
Source: Front Immunol. 2018 May 24;9:1128. doi: 10.3389/fimmu.2018.01128 (PMC5977906; doi:10.3389/fimmu.2018.01128)
Supplement: Supplementary file 2 [file image_2.pdf]

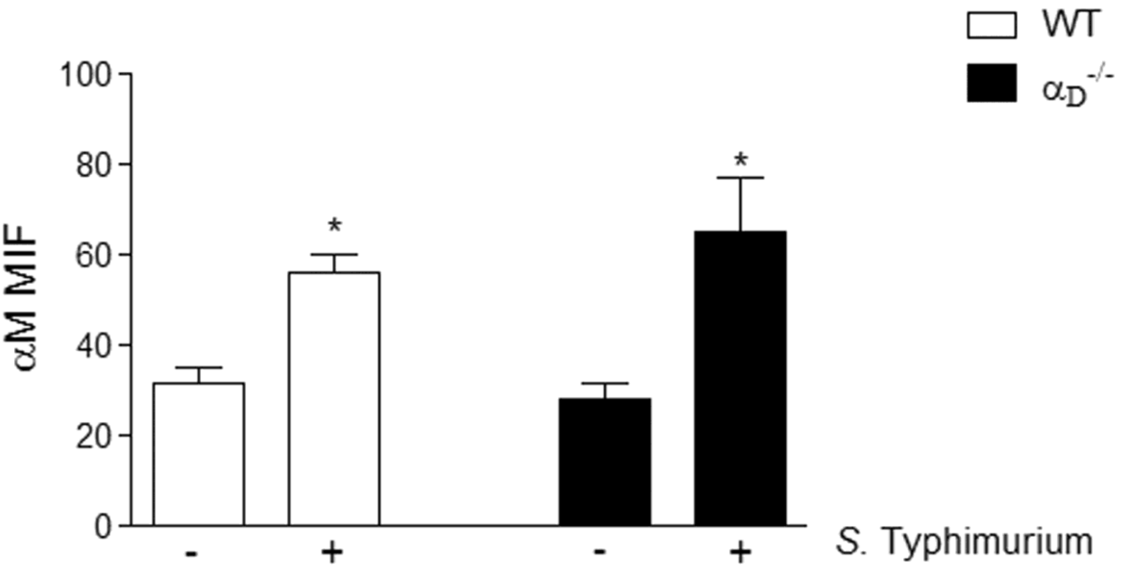

Supplementar Fig. 2 - Salmonella Typhimurium infection increases $\alpha_M\beta_2$ expression in peritoneal cells from WT and $\alpha_D^{-/-}$ mice. WT and $\alpha_D^{-/-}$ mice were infected with *S.* Typhimurium (10^5 CFU/animal) by intraperitoneal injection or were sham-infected with sterile, apyrogenic saline as in Figure 1. After 72h of infection, we collected leukocytes by peritoneal lavage for analysis of $\alpha_M\beta_2$ expression by flow cytometry, as described in "Materials in Methods". The data were represented by the mean fluorescence intensity (MIF). Each bar indicates the mean ± SEM for at least 4 animals. The graphs are representative of two independent experiments. Significant differences ($p < 0.005$) between infected animals and their controls were indicated by asterisks.
