# Supplementary material for: Integrin αDβ2 (CD11d/CD18) Modulates Leukocyte Accumulation, Pathogen Clearance, and Pyroptosis in Experimental Salmonella Typhimurium Infection
Source: Front Immunol. 2018 May 24;9:1128. doi: 10.3389/fimmu.2018.01128 (PMC5977906; doi:10.3389/fimmu.2018.01128)
Supplement: Supplementary file 3 [file image_3.pdf]

A

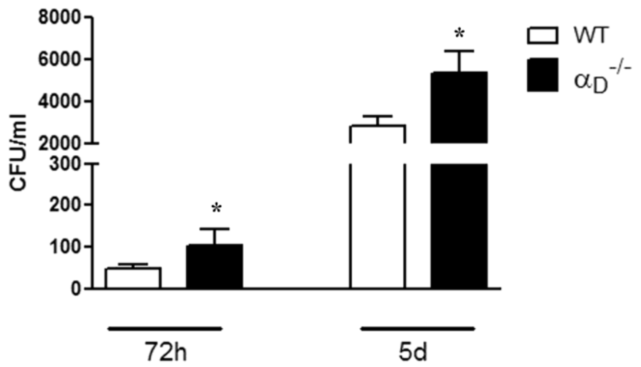

B

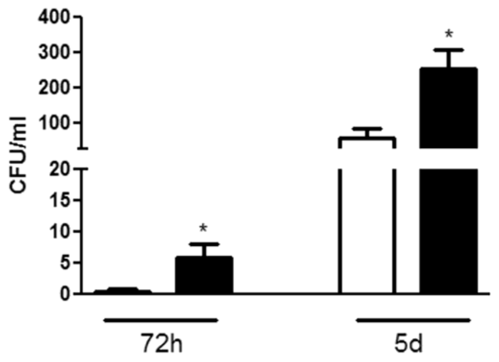

Supplementary Figure 3 -Targeted deletion of integrin $\alpha_D\beta_2$ leads to bacteria increased in the blood and spleen of infected mice.

WT and $\alpha_D^{-/-}$ mice were infected with *S.* Typhimurium (10^5 CFU/animal) by intraperitoneal injection or were sham-infected with sterile, apyrogenic saline as in Figure 1. Blood (A) and spleens (B) were collected 72h, or 5 days later and the number of colony forming units was determined in each sample. Each bar indicates the mean ± SEM of determinations in samples from 5 animals. The data in this figure are representative of three separate experiments. Significant differences ($p < 0.05$) between infected animals and controls are indicated by asterisks.
