# Supplementary material for: Integrin αDβ2 (CD11d/CD18) Modulates Leukocyte Accumulation, Pathogen Clearance, and Pyroptosis in Experimental Salmonella Typhimurium Infection
Source: Front Immunol. 2018 May 24;9:1128. doi: 10.3389/fimmu.2018.01128 (PMC5977906; doi:10.3389/fimmu.2018.01128)
Supplement: Supplementary file 4 [file image_4.pdf]

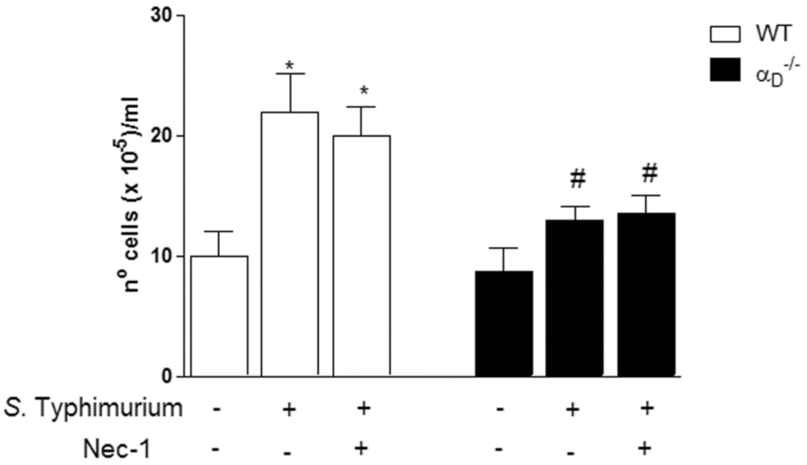

Supplementary Fig. 4 - Administration of Necrostatin-1 *in vivo* did not reverse the pattern of leukocyte accumulation in the peritoneal cavities from *S.* Typhimurium infected mice.

Mice were infected with *S.* Typhimurium as in Figure 1 or were sham-infected and 24 hr later peritoneal fluid was collected and the number of total leukocytes determined. Each bar indicates the mean ± SEM of determinations in samples from ≥ 4 animals. Significant differences ($p \leq 0.05$) between infected and sham-infected mice were indicated by asterisks, and between infected $\alpha_D^{-/-}$ and infected WT mice were indicated by #.
