# Supplementary material for: Integrin αDβ2 (CD11d/CD18) Modulates Leukocyte Accumulation, Pathogen Clearance, and Pyroptosis in Experimental Salmonella Typhimurium Infection
Source: Front Immunol. 2018 May 24;9:1128. doi: 10.3389/fimmu.2018.01128 (PMC5977906; doi:10.3389/fimmu.2018.01128)
Supplement: Supplementary file 5 [file image_5.pdf]

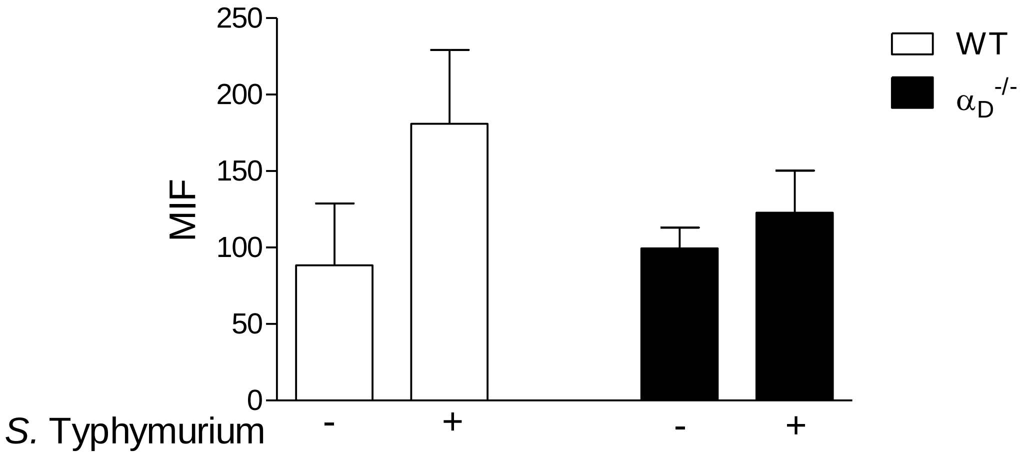


**Supplementary Fig. 5 - Intact production of nitric oxide by cells from $\alpha_D^{-/-}$ *S.* Typhymurium infected mice.** Mice were infected with *S.* Typhimurium as in Figure 1 or were sham-infected. After 72 h of infection, the peritoneal fluid was collected and the cells were labeled with the DAF-FM probe (Molecular Probes) according to the manufacturer's protocol. The data was represented by the mean fluorescence intensity (MIF) obtained by reading on a flow cytometer. Each bar indicates the mean ± SEM for at least 6 animals. Each graph represents the junction of two independent experiments. Significant differences ($p < 0.005$) were indicated by asterisks.
